# Implemented disability-related policies and practices and sustained employment of partially disabled employees: evidence from linked survey and register data

**DOI:** 10.5271/sjweh.4168

**Published:** 2024-09-01

**Authors:** by Raun van Ooijen, Pierre Koning, Cécile RL Boot, Sandra Brouwer

**Affiliations:** 1 Department of Health Sciences, Community and Occupational Medicine, University Medical Center Groningen, University of Groningen, Groningen, The Netherlands; 2 Department of Economics, VU University Amsterdam, Amsterdam, The Netherlands; 3 Department of Public and Occupational Health, Amsterdam UMC, VU University Amsterdam, Amsterdam, The Netherlands; 4 Societal Participation and Health, Amsterdam Public Health Research Institute, Amsterdam, The Netherlands

**Keywords:** disability, employer characteristic, partial disability, The Netherlands, work participation, workplace factor

## Abstract

**Objectives:**

This study examined the associations between implemented disability-related policies and practices (DPP) and sustained employment among partially disabled employees in The Netherlands.

**Methods:**

Employer survey data on implemented DPP were linked to register data on employment outcomes of partially disabled employees (N=6103 employees from N=366 employers). DPP included six domains based on 48 elements: sick leave policy, occupational health and safety services (OHS), prevention policy, reintegration policy, reintegration practices within the current employer and reintegration practices towards another employer. DPP domains were standardized on a 0–1 scale. Separate logistic regression models were estimated for DDP domains on one-year sustained employment adjusted for employee characteristics, firm size, and sector.

**Results:**

Almost all organizations implemented at least one element of DPP on prevention policy, OHS, sick leave policy, and reintegration practices within the current employer, and two-thirds on reintegration policy and reintegration practices towards another employer. Implemented DPP on prevention policy [odds ratio (OR) 2.3, 95% confidence interval (CI) 1.3–4.0], OHS (OR 1.9, 95% CI 1.1–3.2), and sick leave policy (OR 1.8, 95% CI 1.0–3.3) were positively associated with sustained employment. No significant results were found for reintegration policy and both reintegration practices domains. Stratified analysis showed that DDP domains were particularly associated with sustained employment in larger organizations and in the private sector.

**Conclusions:**

Implemented DPP related to sick leave policy, OHS and prevention policy are associated with sustained employment among partially disabled employees, in particular in larger organizations and in the private sector.

Over the past two decades, many OECD countries reformed their sickness and disability benefits programs to encourage sustained employment of partially disabled employees. These reforms were motivated by the notion that many of them have remaining earning capacity and, therefore, can work when well supported by the employer (1, 2). For this purpose, countries lowered disability benefits, tightened the eligibility criteria for full allowances and introduced partial allowances to stimulate combining work and benefits for partially disabled workers. At the same time, stronger responsibilities were introduced for employers to arrange work accommodations and prevent long-term sickness absence and disability benefit receipt. For instance, in The Netherlands, employers are financially incentivized and formally responsible for preventing and managing sick leave and actively facilitating work resumption by providing workplace or task adjustments or searching for other suitable work in or outside the organization.

Although these reforms led to more work resumption and reduced the number of disability benefit applications (3), the prospects of sustained employment have remained low among partially disabled employees. In The Netherlands, about half of the individuals on partial disability benefits and having remaining work capacity are not employed (2). Many of these vulnerable people eventually involuntary exit the labor market (4). As a result, they risk poverty due to considerable reductions in their income when they rely only on partial disability insurance (DI) benefits (2).

Previous research from The Netherlands has shown sizable differences across employers in the prospects of sustained employment of partially disabled employees, even across employers of similar size, workforce composition, and the same sector (5). These employer differences suggest that organizational-related factors can be important in supporting partially disabled employees to continue in paid work. In occupational health research, there is a growing awareness that the organizational context is important in preventing the early labor market exit of workers with poor health (6–8). The organizational context is defined as the characteristics of a workplace, including the physical, psychosocial, and organizational structure of a company (6). As such, both the social interaction between employers and employees and employers’ disability-related policies and practices (DPP) may influence job retention of employees with disabilities (9, 10). So far, only a few studies have examined the relation between DPP and labor market participation. Those studies have particularly focused on the association of DPP on return to work (RTW) (9, 11–14) of sick-listed workers and on reducing disability outcomes at the firm level (12). To date, however, there is limited understanding of the effect of DPP on sustained employment of workers who are awarded partial DI benefits after the sickness period. At the same time, most studies focus on employees’ self-assessments of implemented DPP, which might be biased by memory bias and unawareness of DPP formed within higher organizational levels, such as the human resources department.

Against this background, this study aimed to examine the associations between DPP and sustained employment among partially disabled employees one year after the disability assessment. We considered six DPP domains: sick leave policy, occupational health and safety services, prevention policy, reintegration policy, reintegration practices within the current employer, and reintegration practices towards another employer. We also examined whether there are employer differences (by sector and firm size) in the effect of the six DPP domains on sustained employment.

## Methods

### Institutional context

This study focused on partially disabled employees and their employment status one year after the disability assessment. In The Netherlands, the RTW process comprises two years, which is unique compared to other countries where this period is considerably shorter (16). During this period, employers are actively responsible for facilitating RTW as described in the Gatekeeper Improvement Act (In Dutch: “Wet Verbetering Poortwachter”). Alongside these formal rules, employers are obliged to continue paying wages, incentivizing them to accommodate sick-listed workers and prevent sickness in the first place. After two years of sick leave, for either occupational or non-occupational causes, social insurance legislation (Work and Income Act; WIA) allows employees to apply for a disability benefit.

After a medical disability assessment by an insurance physician and an assessment of the residual earning capacity by a labor expert of the Dutch Employee Insurance Agency (UWV), workers are either awarded full DI benefits when the earnings capacity loss is 80% and above or partial DI benefits when the earnings capacity loss is >35% but <80% of their pre-disability wage earnings. As such, partially disabled workers are deemed to work fewer hours or have jobs with lower hourly wage earnings.

### Study design and data sources

We linked employer-level survey data on implemented DPP to register data covering the sustained employment outcomes of all long-term sick-listed employees who applied for disability benefits and worked for these employers.

**Figure 1:**
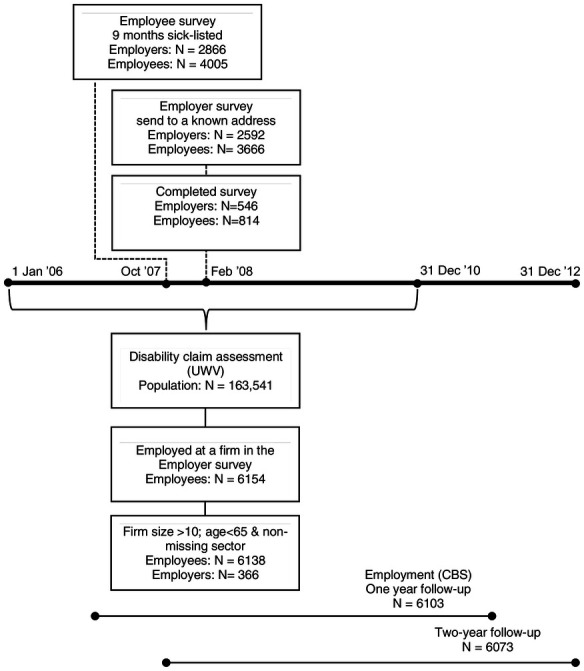
Flowchart of the study population.

Figure 1 shows a flowchart of the selected study population and precise timing of (i) the employer survey, (ii) registry data on DI claim assessments at participating employers, and (iii) one- and two-year employment outcomes of employees at these employers who were assessed for DI benefits. The employer survey is part of the “Pathways into Disability benefits” survey (In Dutch: “Weg naar de WIA”; WNW) that studies the reintegration process of long-term sick-listed workers in The Netherlands (17). The WNW survey was sent in October 2007 to 9-month sick-listed employees who got sick between December 2006 and January 2007 (N=7269). The underlying registry of long-term sick-listed employees is based on mandatory notifications of employers to UWV. In total, 4005 employees participated in the WNW survey. The response rate of 55% is well above the aimed response rate of 40%.

In February 2008, employers (with a known address) of participating employees (N=2592 unique employers of N=3666 employees) were invited to complete a (digital) employer survey. About 21% of the employers (N= 546 unique employers of N=814 employees) completed the employer survey, which was below the aimed response of 33%. This means that an employer survey was effectively collected for 11.2% of the N=7269 invited employees.

The human resources manager (49%), the CEO/manager (21%), or another staff member (15%) usually answered the employer survey. Most employer surveys were completed in the week of the invitation (between 25 and 52 per day). A reminder was sent after one month (and had a paper version of the survey). The last response was received in mid-April 2008. A non-response analysis shows that the characteristics of responding and non-responding employers are very similar in terms of sector and firm size, suggesting that there is no selective response among employers (see supplementary material, URL, table S1).

Register data on disability assessments of long-term sick-listed workers were obtained from the UWV between 2006 and 2010. Longitudinal earnings records on a monthly basis (including hours worked) were obtained from Statistics Netherlands, with employer identifiers that enabled us to link disabled workers to their primary employers who participated in the employer survey. For privacy reasons, individual-level data linkage and statistical analysis were conducted in the microdata environment of Statistics Netherlands using their remote access facilities.

### Sample selection

Our sample for the analysis includes all employees aged 18–64 years who applied for public disability benefits. We included long-term sick-listed employees who were still employed when applying for disability benefits based on employment status four months before the assessment. Employers are financially responsible for workers who were still employed at the moment of the DI claim assessment, during both the period of sick leave and after the disability assessment when they receive disability benefits; and employers pay experience-rated disability benefits premiums. As such, we excluded workers who were not employed four months before the disability assessment. This contains individuals on a temporary contract that ended within the two-years waiting period. In our sample period, employers faced limited financial incentives to organize RTW for these temporary workers. As of 2017, employers also face experience-rated disability benefit premiums for these workers.

We further restricted our sample to workers assessed for DI benefits (at any date) from 1 January 2006 until 31 December 2010, counting about two years in each direction from the employer survey that was fielded at the beginning of 2008. We did so to reduce the possibility that DPP would have been adapted in the meantime. We used the DI benefit assessment date (about two years after the start of sick leave) and not the DI claim date, as the claim date is not in our data set. According to the UWV’s work procedures, the claim date is 4–6 weeks before the assessment date, and the award decision is 8 weeks after the claim date. So, both the claim and award dates are relatively close to the assessment dates covering the period 1 January 2006 until 31 December 2010.

We further excluded persons who were either fully or permanently disabled or assessed as having no loss of earnings capacity. This left us with a sample of 6138 partially disabled employees who applied for a disability benefit after two years of sick leave at 366 employers. For one year and two-year follow-up, we had 6103 and 6073 observations.

### Employee outcome on sustained employment

The outcome of interest for this study was sustained employment, defined as working in a paid job for the same employer or another employer. We considered employment status one year after the disability assessment. For a sensitivity analysis, we also considered employment status after two years and monthly hours worked one year and two years after the assessment.

### DPP measurements

The employer survey provided detailed information on the extent to which organizations implemented DPP originating from 48 elements. These elements were divided over six DPP domains: (i) sick leave policy, N=8; (ii) occupational health and safety services (OHS), N=6; (iii) prevention policy, N=8; (iv) reintegration policy, N=8; (v) reintegration practices within the current employer, N=11; and (vi) reintegration practices towards another employer, N=7.

Sick leave policy outlines the procedures and conditions under which organizations handle sick leave. It includes a sick leave protocol, procedures for monitoring sick leave, a division of responsibilities, guidelines on communication, and measures to control sick leave. In The Netherlands, every employer must contract an OHS or occupational physician and can acquire services, including a risk inventory and evaluation, periodic occupational health examinations, working conditions consultations, and supervision of sick leave by an occupational physician or case manager. Prevention policies contain measures to prevent sick leave, including a risk inventory and evaluation, periodic occupational health examinations, consulting a health and safety coordinator, and programs to reduce stress and physical workload. Reintegration policies contain the steps and guidelines for supporting work resumption in case of long-term sick leave. The employer and employee are obliged to make an RTW plan containing reintegration practices to facilitate work resumption in their own or other work. These practices may include reduced work hours, workplace adaptations, modified job duties, or job changes. After one year, the process is evaluated, and for the second year of sick leave, the plan should include reintegration practices towards another employer, such as registration at an employee agency or vocational education or training.

For each DDP domain, the employer was asked to state: “Which existing elements are included?” See Box 1 for details. A factor analysis using principal component analysis extraction was conducted on these 48 items, resulting in six factors representing the DPP domains of the survey. Cronbach’s alphas ranged from 0.58 (OHS) to 0.89 (reintegration policy), indicating acceptable (moderate to high) internal reliability of the underlying items.

Box 1 **Implemented disability-related policies and practices (DPP) items (yes/no) from the employer survey per element of the disability management process. For each DDP domain, the employer was asked to state: “Which existing elements are part of the DDP domain?”**

**Table:** 

**SICK LEAVE POLICY (N=8)**
1. Absenteeism protocol
2. Division of responsibilities
3. Guidelines on return-to-work interviews
4. Training for supervisors to conduct return-to-work interviews
5. Monitoring and analyzing absenteeism
6. Guidelines on how to communicate the absenteeism policy
7. Measures to control absenteeism and to identify causes of absenteeism
8. Periodic review of policies and practices
**OCCUPATIONAL HEALTH AND SAFETY SERVICES (N=6)**
9. Conducting Risk Inventory and Evaluation (RI&E)
10. Offering working conditions consultation hours for employees
11. Offering voluntary Periodic Occupational Health examinations
12. Absence supervision (by an Occupation Physician)
13. Psychological counseling
14. Workplace surveys
**PREVENTION POLICY (N=8)**
15. Regularly conducting Risk Inventory and Evaluation or voluntary Periodic Occupational Health examinations
16. Following up measures from the Risk Inventory and Evaluation
17. Communication about workloads with employees
18. Health and safety coordinator
19. RSI prevention program
20. Program to reduce stress in the workplace
21. Program to reduce the physical workload
22. Other preventive programs, e.g., in-company fitness, relaxation exercises, anti-smoking, and confidential advisors
**REINTEGRATION POLICY (N=8)**
23. Protocol for long-term absence and job reassignment
24. Division of responsibilities in case of long-term absence and job reassignment
25. Guidelines on supporting return-to-work
26. Training for supervisors to manage return-to-work/job reassignment
27. Establishing a committee to find alternative jobs in the organization for long-term sick-listed employees
28. Communicating reintegration measures/job reassignment to sick-listed workers
29. Measures to facilitate work resumption in own work or other work, e.g. job rotation, teleworking
30. Periodic review of policies and practices
**REINTEGRATION PRACTICES WITH THE CURRENT EMPLOYER (N=11)**
31. Reduced work hours
32. Adaptions in the workplace
33. Modified job duties (temporal/permanent)
34. Flexible work hours (temporal/permanent)
35. Individual counseling/coaching
36. Return to work on a therapeutic basis
37. Training and Education
38. Reimbursement of therapy or treatment
39. Job change/reassignment
40. Temporarily work resumption at another employer
41. Waiting list mediation
**REINTEGRATION PRACTICES WITH ANOTHER EMPLOYER (N=7)**
42. Outplacement trajectory
43. Registration at an employee agency
44. Actively searching for suitable vacancies at other companies
45. Notification in an inter-organizational cooperation in return-to-work and labor market reintegration
46. Vocational education or training to resume work at another employer
47. Counseling or coaching by an external reintegration company
48. Counseling or coaching by another employer

Employers were asked to indicate whether each DPP element was implemented within their organization (yes/no). For each DPP domain, the percentage of organizations that implemented minimally one of the domain-specific elements was computed, together with the corresponding mean number and standard deviation (SD). To enhance the comparability of the six DPP domains, we summed the underlying elements and normalized the scales between 0 and 1 by subtracting the minimum and dividing by the range (maximum-minimum). As a result, for each DPP domain, organizations with the most comprehensive policies and practices have a standardized value of 1, and organizations with the least comprehensive ones have a value of 0.

### Covariates

All covariates were obtained from the register data from Statistics Netherlands (sector and firm size on the employer level and age and gender at the employee level) and the UWV (educational attainment, primary diagnosis, comorbidities, and year of disability application, all at the employee level). The sector was coded at the time of the DI benefits assessment and based on the International Standard Industrial Classifications (ISIC). We distinguished between five main sectors: (i) agriculture, construction and manufacturing; (ii) trade, transportation, recreation and support service; (iii) financial and professional services; (iv) public administration and education; and (v) health. Firm size coded at the time of DI benefits assessment and was categorized into six groups to address a potential non-linear relationship: 10–49, 50–249, 250–499, 500–999, 1000–4999, ≥5000 employees. Age was coded at the time of the DI benefits assessment based on the month of birth and categorized into eight groups: 18–29, 30–34, 35–39, 40–44, 45–49, 50–54, 55-59, and 60–64 years. Educational attainment (highest level) was documented at the DI benefit assessment and classified into four groups according to the International Standard Classification of Education (ISCED 2011): tertiary (bachelor’s or higher), upper secondary (pre-university, general secondary, or senior secondary vocational education), lower secondary (pre-vocational secondary education), and primary education, including persons who did not complete any education. For administrative reasons, educational attainment was missing for a small part of the sample, for which we included a missing category. Primary diagnosis was documented at the DI benefit assessment and based on diagnosis codes from the World Health Organization's International Classification of Diseases, Tenth Revision (ICD-10). We distinguished between the six most prevalent disease categories: mental disorders, musculoskeletal disorders, cancers, circulatory disorders, neurological disorders, and other causes of disability (Chapters II, V, VI, IX, XIII, and all other chapters of ICD-10). Having comorbidities was defined as the presence of either one or more diseases in a different disease category (ICD-10 chapter) co-occurring with the primary diagnosis.

### Statistical model

Multilevel logistic regression analyses at the organizational level were used to examine the extent to which the separate DPP domains were associated with sustained employment. Hence, we estimated separate models for each DPP domain to avoid multicollinearity problems due to the correlation between the scores of the six different DPP (see supplementary table S2). For each DPP domain, we estimated logistic regression models with and without adjusting for sector, firm size, age, gender, educational attainment, primary diagnosis, comorbidities, and year of DI assessment. Next, we tested for interactions between each DPP domain and firm size and sector by conducting stratified analyses. We distinguished between smaller and larger organizations (by dividing the sample in half) and between public and private sector organizations. Finally, we performed sensitivity analyses to consider whether the associations remained similar at the two-year follow-up and when using monthly hours worked instead of employment Also, we examined all DDP domains simultaneously in one model. All analyses were conducted using Stata/SE version 16 for Windows (StataCorp LLC, College Station, TX, USA). Standard errors were clustered on the employer level. The level of significance was set at P<0.05.

## Results

Table 1 shows the descriptive statistics for the study sample. The mean age of the employees in the sample was 47.6 (SD 9.3) years, 61.7% was female. Musculoskeletal (25.6%) and mental disorders (27.6%) were the most often diagnosed disease groups. In total, 59.5% of the sample worked in the public sector, with 32.1% in public administration and education and 27.4% in health care. Most employees worked in larger organizations with >1000 employees: 51.3% in organizations with 1000–4999 employees and 33.3% in organizations with >5000 employees. One year after the disability assessment, 58.6% of the partially disabled employees continued in paid employment. Older employees, women, employees diagnosed with nervous, circulatory or mental disorders and working in the health, financial and professional service sector were less likely to continue in paid employment. Employees with higher levels of education and working in larger companies were more likely to continue in paid employment. Two years after the disability assessment, 53.9% of the partially disabled employees were in continued paid employment. Having a similar distribution across groups as the one-year time point.

**Table 1:** Characteristics of the study population and sustained employment one and two years after the disability assessment.

		Total % (N=6103)	% employed after one year (N=6103)	% employed after two years (N=6073)
Total		100	58.6	53.9
Age				
	18–29	4.8	60.1	60.1
	30–34	6.4	64.3	63.0
	35–39	9.3	63.5	60.6
	40–44	13.6	61.3	58.0
	45–49	16.5	64.5	60.2
	50–54	21.6	56.7	52.6
	55–59	21.4	54.7	46.3
	60–64	6.4	40.9	31.2
Gender				
	Men	38.3	60.6	54.8
	Women	61.7	57.3	53.3
Education				
	Primary	18.8	45.8	41.0
	Lower secondary	23.3	53.4	49.9
	Upper secondary	27.1	64.1	59.4
	Tertiary	14.8	64.9	59.9
	Missing	16.1	65.7	59.4
Primary diagnosis				
	Cancer	9.3	65.6	58.1
	Mental	27.6	56.4	51.3
	Nervous	6.9	51.6	45.5
	Circulatory	9.3	52.9	47.7
	Musculoskeletal	25.6	59.1	56.6
	Injury	5.7	64.1	63.1
	Other	13.1	60.5	54.9
	Missing	2.5	68.2	58.2
Comorbidity				
	Yes	37.0	53.9	48.9
	No	63.0	61.3	56.8
Sector				
	Agriculture, construction and manufacturing	6.4	58.3	55.9
	Trade, transportation, recreation and support services	20.2	60.3	56.3
	Financial and professional services	13.9	55.3	47.4
	Public administration and education	32.1	63.6	56.9
	Health	27.4	53.1	51.4
Firm size				
	10–49	0.8	56.3	64.6
	50–249	3.4	53.1	49.5
	250–499	4.1	60.8	54.0
	500–999	7.1	57.7	53.7
	1000–4999	51.3	55.7	51.9
	5000>	33.3	63.5	57.1
Year of assessment				
	2006	13.9	60.5	55.3
	2007	18.6	60.2	54.8
	2008	21.1	58.5	53.3
	2009	20.7	55.3	53.0
	2010	25.8	59.1	53.6

Table 2 shows the descriptive statistics of the implemented DPP for each domain. Most organizations implemented at least one DPP element for the DPP domains sick leave policy (98.5%), occupational health and safety services (98.9%), prevention policy (99.8%), and reintegration practices within the current employer (93.1%). For the domains of reintegration policy and reintegration practices towards another employer, two-thirds of the employers implemented at least one DPP element. For reintegration policy, there are significant differences across firm size, with higher percentages in larger organizations (80%) than in smaller organizations (56%). For reintegration practices towards another employer, there are significant differences across sectors, with higher percentages in private organizations (75%) than in public organizations (60%).

**Table 2:** Descriptive statistics of the implemented elements of the disability-related policies and practices (DPP) domains for the total sample and stratified by sector and firm size, (N=6103). [OHS=occupational health & safety services; RTW=return-to-work; SD=standard deviation.]

DPP domain	Elements	Number of implemented elements					Standardized score	
		% ^a^	Mean		SD		Mean	SD
Sick leave policy	8	98	6.5		1.8		0.72	0.20
OHS	6	99	3.6		1.8		0.52	0.26
Prevention policy	8	100	5.1		2.0		0.57	0.22
Reintegration policy	8	68	3.1		2.7		0.39	0.33
RTW practices current employer	11	93	6.8		3.0		0.62	0.27
RTW practices another employer	7	66	1.9		1.8		0.24	0.23
	Number of implemented elements							
	Private (N=2464)				Public (N=3639)			
	%	Mean	SD		%		Mean	SD
Sick leave policy	97	6.6	2.1		99		6.4	1.6
OHS	100	4.2	1.9		98		3.2	1.6
Prevention policy	100	5.5	2.2		100		4.9	1.7
Reintegration policy	65	3.1	2.8		69		3.1	2.6
RTW practices current employer	90	6.3	3.3		95		7.2	2.7
RTW practices another employer	75	2.2	1.8		60		1.8	1.8
	Number of implemented elements							
	Large companies (N=3069)				Small companies (N=3034)			
	%	Mean	SD		%		Mean	SD
Sick leave policy	100	6.8	1.6		97		6.2	1.9
OHS	100	4.2	1.9		98		3.0	1.5
Prevention policy	100	5.5	2.2		100		4.8	1.7
Reintegration policy	80	3.5	2.5		56		2.7	2.8
RTW practices current employer	94	6.8	2.8		92		6.8	3.1
RTW practices another employer	65	2.0	1.8		67		1.9	1.8

^a^ % at least one implemented element within the DDP domain.

### Associations between DPP domains and sustained employment

Table 3 presents the results of the unadjusted and adjusted logistic regression models for the standardized DDP scores. In the unadjusted models, prevention policy (OR 2.4, 95% CI 1.1–5.4) and OHS (OR 2.3, 95% CI 1.1–4.7) were positively associated with sustained employment. In the adjusted models, most of the coefficients had similar magnitudes (<10% difference) compared to the unadjusted models. In the adjusted models, sick leave policy (OR 1.8, 95% CI 1.0–3.3), OHS (OR 1.9, 95% CI 1.1–3.2), and prevention policy (OR 2.3, 95% CI 1.3–4.0) were positively associated with sustained employed. No significant associations were found for reintegration policy and reintegration practices within the current or towards another employer.

**Table 3:** Implemented disability-related policies and practices (DPP) domains and sustained employment, one and two years after the assessment. Odds ratios (OR) and 95% confidence interval (CI) from multilevel logistic regression. [OHS=occupational health & safety services; RTW=return-to-work]

DPP domain	One year (N=6103)								Two years (N=6073)		
	Unadjusted			Adjusted			Unadjusted			Adjusted	
	OR	95% CI		OR ^a^	95% CI		OR	95% CI		OR ^a^	95% CI
Sick leave policy	1.88	0.82–4.28		1.80	0.97–3.34		1.60	0.69–3.73		1.80	0.99–3.26
OHS	2.27	1.09–4.70		1.85	1.08–3.16		1.84	0.85–3.99		1.72	1.03–2.89
Prevention policy	2.38	1.05–5.42		2.30	1.32–4.02		1.91	0.78–4.69		1.91	1.07–3.39
Reintegration policy	1.34	0.79–2.29		1.33	0.91–1.96		1.29	0.76–2.17		1.29	0.89–1.86
RTW practices current employer	1.68	0.91–3.10		1.52	0.95–2.42		1.53	0.85–2.76		1.46	0.94–2.25
RTW practices another employer	1.50	0.64–3.51		1.53	0.86–2.72		1.60	0.71–3.60		1.52	0.87–2.64

^a^ Models adjusted for age, gender, educational attainment, primary diagnosis, comorbidity, sector, firm size, and year indicator. Standard errors are clustered on the employer level.

### Employer differences in implemented DPP domains for sustained employment

Testing for interactions between each DPP domain and sector (public versus private) and firm size (small versus large) revealed significantly higher associations for employees working in the private sector and for employees working in larger companies only (table 4). No statistically significant associations were found for employees working in the public sector and smaller companies. For employees in private organizations, additionally, more comprehensive RTW policies and practices with another employer were significantly associated with sustained employment.

**Table 4:** Implemented disability-related policies and practices (DPP) domains and sustained employment one year after the disability assessment stratified by sector and firm size. Odds ratios (OR) and 95% confidence interval (CI) from multilevel logistic regression. [OHS=occupational health & safety services; RTW=return-to-work]

DPP domain	Unadjusted						Adjusted				
	Public (N=3639)			Private (N=2464)			Public (N=3639)			Private (N=2464)	
	OR	95% CI		OR	95% CI		OR ^a^	95% CI		OR ^a^	95% CI
Sick leave policy	1.33	0.76–2.32		2.50	0.65–9.60		1.40	0.82–2.41		1.58	0.70–3.58
OHS	1.39	0.84–2.28		4.60	1.50–14.09		1.10	0.69–1.63		2.85	1.26–6.46
Prevention policy	1.29	0.74–2.25		4.36	1.35–14.07		1.46	0.92–2.32		3.17	1.68–5.96
Reintegration policy	0.83	0.63–1.10		2.49	1.02–6.08		0.96	0.72–1.28		2.22	1.33–3.71
RTW practices current employer	1.27	0.81–1.98		2.27	0.68–7.58		1.23	0.83–1.82		1.78	0.89–3.56
RTW practices another employer	0.71	0.48–1.05		4.97	1.39–17.71		0.86	0.54–1.36		2.73	1.29–5.75
	Large (N=3069)			Small (N=3034)			Large (N=3069)			Small (N=3034)	
	OR	95% CI		OR	95% CI		OR ^a^	95% CI		OR ^a^	95% CI
Sick leave policy	3.46	0.72–16.52		1.18	0.75–1.86		3.00	1.08–8.29		1.14	0.71–1.84
OHS	4.06	1.38–11.90		1.08	0.71–1.65		3.27	1.32–8.07		0.91	0.61–1.36
Prevention policy	3.94	1.43–10.84		1.03	0.62–1.73		3.28	1.72–6.27		1.19	0.75–1.90
Reintegration policy	1.98	0.78–4.98		0.97	0.73–1.29		1.72	0.95–3.10		1.02	0.76–1.36
RTW practices current employer	2.41	0.67–8.69		1.25	0.84–1.87		1.82	0.78–4.24		1.23	0.86–1.75
RTW practices another employer	2.50	0.63–9.92		0.90	0.63–1.31		2.30	0.81–6.51		1.04	0.71–1.52

^a^ Models adjusted for age, gender, educational attainment, primary diagnosis, comorbidity, sector, firm size, and year indicator. Standard errors are clustered on the employer level.

### Sensitivity analyses

The sensitivity analyses for sustained employment two years after the disability assessment yielded similar results compared to the results obtained for the one-year follow-up period (table 3). Also, the results for monthly hours of work were very similar to the results for sustained employment, both in relative terms and statistical significance (which is, for some variables, higher) (see supplementary table S3). For the model where we included the DPP domains simultaneously, the estimates are, as expected, somewhat lower than for the separate estimations. The prevention policy and sick leave policy domains were no longer statistically significant. The OHS domain remained statistically significant (see supplementary table S4).

## Discussion

The findings of this study showed that different degrees of the implementation of DPP related to prevention of sick leave, OHS and sick leave policy are associated with sustained employment among partially disabled employees. No significant results were found for DPP related to reintegration policy and RTW practices within the current or towards another employer. The results were very similar when using monthly working hours as the outcome variable, both in relative terms and statistical significance. Also, the results were similar for the one- and two-year follow-up period, suggesting that DPP have persistent effects on the sustained employment of partially disabled employees. We believe that these effects hold beyond the two-year follow-up period, as if partially disabled workers were unable to meet minimal performance expectations (with potential accommodation), measures and decisions regarding work continuation would likely been taken by the employer within the first year.

When considering differences by sector, we found that all implemented DPP domains except sick leave policy and RTW practices within the current employer contributed to sustained employment for workers in private organizations but not for workers in public organizations. In addition, prevention policy, OHS and sick leave policy contributed to sustained employment for workers in larger organizations but not for workers in smaller organizations.

For the interpretation of the effect sizes, it is important to stress that we used standardized scores to enhance the comparability of the six DPP domains. We summed the underlying elements and normalized the scales between 0 and 1, rendering scores that are informative on the extent to which these policies are used. To interpret the size of the effect of the respective DPP on sustained employment, however, it is important to bear in mind that the actual variation in scores may differ across the DPP. Specifically, for the DPP sick leave policy, OHS and prevention policy this implies an increase of 1 SD in the score of corresponding implemented DPP is associated with about a 20–25 percentage point higher likelihood of sustained employment. The importance of policies and practices related to sick leave is in line with existing research on long-term sick leave prevention programs (18). It also supports recent OECD recommendations for the need for early and active intervention of employers to bring sick-listed workers back to work as soon as possible and to react quickly when a return to the same job, or the same employer, is not possible (2). According to the OECD, this involves active management and monitoring of sickness absence, such as in The Netherlands, where the sickness program plays a key role in gatekeeping entry to disability benefits.

We did not find significant associations between the DPP domains reintegration policy, RTW practices toward the current and RTW practices towards another employer in the total sample. However, addressing our second research aim, we found that both reintegration policy and RTW practices toward another employer are associated with sustained employment for partially disabled employees in private organizations. This may be due to the differences in implemented DPP.

Most organizations implemented a range of measures in the DPP domains sick leave policy, OHS, prevention policy, and RTW practices within the current employer. This holds for two-thirds of the organizations for the DPP domains reintegration policy and RTW practices towards another employer. When considering private organizations, the percentage of employers reporting implemented elements of RTW practices towards the current employer (75%) is higher than in public organizations (60%). Yet, for reintegration policy, the percentage is very similar in public and private organizations.

The difference in the results between smaller and larger companies might be due to available resources. Smaller companies most likely have small (or no) human resource teams and a smaller budget. Consequently, they can offer less when it comes to the development, implementation, and management of DPP. Further, they rely more on external health and safety services and reintegration providers, which may make supervisors less involved. Still, we found that the number of implemented DPP are only slightly lower in smaller companies. It is only for the reintegration policy domain that the differences are more substantial between smaller and larger companies. Another explanation could be that smaller companies have fewer opportunities for sustained employment than larger organizations, making DPP less effective.

Our study contributes to a growing literature that links longitudinal register data to employer surveys to study the contribution of employer management and human resource practices to employees’ work outcomes (18–20). No previous study exists that focuses on the effects of DPP on sustained employment of partially disabled workers. However, the importance of DPP for sustained employment of this vulnerable group of workers is in line with earlier studies on workplace interventions and RTW programs (21).

### Strengths and limitations

The strength of this study concerns the large and representative study population and the ability to link the employer survey to the individual-level longitudinal registry data. Alongside this, the survey contains very detailed, semi-objective data on the use of DPP. While the employer survey was fielded in 2008, the unique set-up with combined registry data allows us to examine whether a firm’s DPP can promote sustained employment while controlling for a large set of employee and firm characteristics, including diagnosis, co-morbidities, education, sector, and firm size. The results can be extended to the current situation as long-term sick leave and disability policies and the activating role of the employer remained similar to when the survey was fielded, as no major sick leave and disability policy reforms took place. The findings are particularly relevant due to rising long-term sick leave and disability applications.

Though there was no evidence of selective non-response based on sector and firm size, only about 20% of the contacted employers participated in an employer survey. While this falls within the usual bandwidth of organizational research (22), it could be that organizations with the most comprehensive DDP were more inclined to fill in the survey. Still, for our sample of employers, we found substantial variation in the number of implemented DPP, which we found to be associated with sustained employment among partially disabled employees for the DPP related to sick leave policy, OHS, and prevention of sick leave. Another limitation was that the survey contained answers on DPP from one staff member who might be biased regarding its content and might not oversee how it manifests inside the organization. Particularly in larger organizations, the content of DPP might vary across divisions. Also, the employer survey only measured the implementation of DPP and not the quality of the underlying measures.

### Concluding remarks

Our study showed that implemented DPP related to sick leave policy, occupational health and safety services, and prevention of sick leave are associated with sustained employment among partially disabled employees. Moreover, partially disabled workers in larger organizations and the private sector seem to be at a lower risk for labor market exit than those in smaller and public organizations. Encouraging organizations to invest more in implementing prevention and reintegration policies and practices may result in better employment opportunities for this vulnerable group of employees.

## Supplementary material

Supplementary material
