# Supplementary material for: Implemented disability-related policies and practices and sustained employment of partially disabled employees: evidence from linked survey and register data
Source: Scand J Work Environ Health. 2024 Aug 30;50(6):437–46. doi: 10.5271/sjweh.4168 (PMC11391366; doi:10.5271/sjweh.4168)
Supplement: Supplementary material [file SJWEH-50-437-S001.pdf]

Implemented disability-related policies and practices and sustained employment of partially disabled employees: evidence from linked survey and register data[1]

by Raun van Ooijen, PhD,[2] Pierre Koning, PhD, Cécile RL Boot, PhD, Sandra Brouwer, PhD

1. *Supplementary tables*
2. *Correspondence to: Raun van Ooijen, University Medical Center Groningen, Department of Health Sciences, University of Groningen, PO-box 30001, 9700 RB Groningen, Groningen, The Netherlands. [E-mail: r.van.ooijen@umcg.nl]*

Table S1. Firm characteristics among firms that responded or not responded to the employer survey.

	Not responded Percent %	Responded Percent %
**Sector** (Chi2(4) = 13.1, P=0.011)		
Agriculture, construction and manufacturing	17.2	16.1
Trade, transportation, recreation and support services	24.2	19.8
Financial and professional services	17.4	16.6
Public administration and education	20.0	22.0
Health	21.2	25.5
**Firm size** (Chi2(5) = 2.2, P=0.818)		
<10	12.0	12.2
11-25	10.1	10.1
26-50	9.1	8.1
51-100	9.3	9.2
101-250	10.9	12.5
250>	48.6	48.0

Table S2. Correlation coefficients of the implemented Disability-related Policies and Practices domains (N=6103).

	Sick leave policy	OHS	Prevention policy	Reintegration policy	Reintegration practices current employer	Reintegration practices another employer
Sick leave policy	1					
OHS	0.26 [a]	1				
Prevention policy	0.54	0.39	1			
Reintegration policy	0.42	0.20	0.54	1		
Reintegration practices current employer	0.46	0.09	0.45	0.45	1	
Reintegration practices another employer	0.28	0.22	0.44	0.46	0.42	1

[a] All the correlation coefficients are statistically significant at the 1% level.

Table S3. Implemented Disability-related Policies and Practices domains and monthly hours worked, one and two years after the assessment. OLS Regression estimates (b) and 95% confidence interval (CI) from multilevel logistic regression.

DPP domain	One year (N=6103)				Two years (N=6073)			
	Unadjusted		Adjusted		Unadjusted		Adjusted	
	b	95% CI	b[a]	95% CI	b	95% CI	b[a]	95% CI
Sick leave policy	32.1	-4.2-68.5	27.1	6.8-47.3	27.7	-7.4-62.7	27.0	8.9-45.0
OHS	37.2	5.7-68.7	19.5	0.6-38.5	29.7	-2.6-61.9	16.6	-1.1-34.4
Prevention policy	36.8	-1.3-74.9	33.1	13.8-52.4	29.0	-10.1-68.2	26.3	7.2-45.4
Reintegration policy	13.0	-11.2-37.1	15.6	2.9-28.3	11.8	-11.1-34.7	14.8	3.2-26.4
RTW practices current employer	16.8	-11.0-44.6	16.1	-0.2-32.5	12.6	-13.5-38.7	13.2	-1.6-27.9
RTW practices another employer	15.2	-24.6-55.1	19.7	-0.1-39.5	16.5	-20.4-53.4	17.1	-1.5-35.7

[a] Models adjusted for age, gender, educational attainment, primary diagnosis, comorbidity, sector, firm size, and year indicator. Standard errors are clustered on the employer level.

Table S4 Implemented Disability-related Policies and Practices (DPP) domains and sustained employment, one and two years after the assessment. All DPP included in one model. Odds (OR) ratios and 95% confidence interval (CI) from multilevel logistic regression.

DPP domain	One year				Two years			
	Unadjusted		Adjusted		Unadjusted		Adjusted	
	OR	95% CI	OR[a]	95% CI	OR	95% CI	OR[a]	95% CI
Sick leave policy	1.02	0.60-1.75	1.14	0.68-1.93	1.00	0.60-1.67	1.28	0.76-2.18
OHS	2.03	1.14-3.62	1.61	1.02-2.53	1.68	0.94-2.99	1.54	1.0-2.37
Prevention policy	1.50	0.79-2.86	1.65	0.89-3.06	1.24	0.66-2.30	1.28	0.70-2.34
Reintegration policy	0.96	0.70-1.31	1.03	0.75-1.42	0.97	0.74-1.26	1.03	0.77-1.37
RTW practices current employer	1.44	0.84-2.45	1.21	0.8-1.84	1.32	0.81-2.13	1.19	0.82-1.74
RTW practices another employer	0.90	0.55-1.47	0.94	0.6-1.49	1.14	0.69-1.86	1.01	0.64-1.59

[a] Models adjusted for age, gender, educational attainment, primary diagnosis, comorbidity, sector, firm size, and year indicator. Standard errors are clustered on the employer level.
